# Epicardial Adipose Tissue Is Nonlinearly Related to Anthropometric Measures and Subcutaneous Adipose Tissue

**DOI:** 10.1155/2015/456293

**Published:** 2015-06-01

**Authors:** Miroslav Šram, Zvonimir Vrselja, Igor Lekšan, Goran Ćurić, Kristina Selthofer-Relatić, Radivoje Radić

**Affiliations:** ^1^Department of Cardiology, Clinic of Internal Medicine, Osijek University Hospital Centre, 31000 Osijek, Croatia; ^2^Department of Anatomy and Neuroscience, Faculty of Medicine, University of J.J. Strossmayer in Osijek, 31000 Osijek, Croatia; ^3^Department of Cardiac Surgery, Clinic of Surgery, Osijek University Hospital, 31000 Osijek, Croatia; ^4^DNA Laboratory, Department of Chemistry, Biochemistry and Clinical Chemistry, Faculty of Medicine, University of J.J. Strossmayer in Osijek, 31000 Osijek, Croatia; ^5^Department of Internal Medicine, Faculty of Medicine, University of J.J. Strossmayer in Osijek, 31000 Osijek, Croatia

## Abstract

*Introduction*. Adipose tissue is the largest endocrine organ, composed of subcutaneous (SAT) and visceral adipose tissue (VAT), the latter being highly associated with coronary artery disease (CAD). Expansion of epicardial adipose tissue (EAT) is linked to CAD. One way of assessing the CAD risk is with low-cost anthropometric measures, although they are inaccurate and cannot discriminate between VAT and SAT. The aim of this study is to evaluate (1) the relationship between EAT thickness, SAT thickness and anthropometric measures in a cohort of patients assessed at the cardiology unit and (2) determine predictive power of anthropometric measures and EAT and SAT thickness in establishment of CAD. *Methods*. Anthropometric measures were obtained from 53 CAD and 42 non-CAD patients. Vascular and structural statuses were obtained with coronarography and echocardiography, as well as measurements of the EAT and SAT thickness. *Results*. Anthropometric measures showed moderate positive correlation with EAT and SAT thickness. Anthropometric measures and SAT follow nonlinear *S curve* relationship with EAT. Strong nonlinear *power curve* relationship was observed between EAT and SAT thinner than 10 mm. Anthropometric measures and EAT and SAT were poor predictors of CAD. *Conclusion*. Anthropometric measures and SAT have nonlinear relationship with EAT. EAT thickness and anthropometric measures have similar CAD predictive value.

## 1. Introduction

The most common chronic disorder nowadays is obesity, which is defined as excessive accumulation of adipose tissue (fat), traditionally defined with body mass index (BMI) exceeding 30 kg/m^2^. The majority of adipose tissues in the human body are deposited as subcutaneous or visceral fat, differing in their structure and function [1]. Subcutaneous adipose tissue (SAT) is primarily located on the extremities, while visceral adipose tissue (VAT) is located around internal organs. Adipose tissue has important endocrine role, as genetic analyses have shown that adipose tissue (especially VAT) expresses numerous secretory proteins (adipocytokines) [2]. Recently it was established that proinflammatory adipocytokines secreted by thickened epicardial adipose tissue (EAT), VAT located around the heart and coronary arteries, lead to the development of CAD [3, 4]. Obesity is recognized as risk factor for coronary artery disease (CAD) [5, 6]. Particularly risk-increasing is abdominal obesity, characterized with predominantly abdominal accumulation of fat (VAT) [7, 8]. Abdominal obesity is key characteristic for establishment of the metabolic syndrome, together with any two of the following: hypertriglyceridemia, reduced high density lipoprotein (HDL), raised blood pressure, and disorders of carbohydrate metabolism (raised fasting plasma glucose or diabetes type 2) [9]. Since BMI is general indicator of obesity, measures of abdominal obesity, such as waist circumference (WC), waist-to-hip ratio (WHR), and waist-to-height ratio (WHR), are suggested as more accurate in describing the distribution of body fat compared. Ultrasound anthropometric indicators, such as SAT and EAT thickness, have been recently proposed as methods of assessment of body fat distribution. Research on relationships between “classic” and ultrasound anthropometric indicators is limited [10].

Coronary heart disease is the leading cause of death worldwide. Coronary angiography is the criterion standard for detecting significant flow-limiting stenosis and direct imaging of atherosclerotic changes in coronary arteries. Because of the inherent limitations, disadvantages and complications of coronary angiography, attention has been directed toward using physiologic, noninvasive modalities to determine the severity of coronary stenosis. Epicardial adipose tissue thickness has been reported as a marker for the presence and severity of coronary artery disease [3, 4, 11], superior to WC and WHR.

Due to limited research in the field, we performed the detailed analysis of the relationship between anthropometric measures (WC, hip circumference (HC), WHR, BMI, and waist-to-height ratio) and echocardiographically obtained EAT and SAT thickness in a cohort of patients admitted at the cardiology unit. We also tested power of these “classical” and ultrasound anthropometric measures for the prediction of CAD.

## 2. Methods

### 2.1. Study Population

In this study 95 Caucasian subjects were included, 55 males and 40 females, all of whom signed an informed consent. Subject's age spanned from 31 to 80 years, with BMI of 19.9 to 38.7 kg/m^2^. Due to clinical symptoms all subjects underwent echocardiography and coronarography at the Cardiology Department of Clinical Hospital Center Osijek, Croatia. Exclusion criteria were diabetes and chronic renal failure.

### 2.2. Epicardial and Subcutaneous Adipose Tissue Thickness Measurement

Ultrasound measurements of EAT thickness were performed with Siemens Acuson V70 and linear probe L10 (5–11 MHz) and SAT thickness with probe P4-2 (all Siemens Medical Solutions, Malvern, PA, USA). EAT thickness was assessed using M mode in long parasternal axis with subject in left lateral decubitus position. The EAT was identified as echo-free space between the myocardial wall and the visceral layer of pericardium in end-diastole above right ventricle [3, 4, 11, 12]. Subcutaneous adipose tissue thickness was measured above umbilicus while the subject was on his/her back. All measurements were done in triplicate with probe repositioning by one sonographer.

### 2.3. Anthropometry

Anthropometric measures were taken in all subjects in standing position, while being barefoot on a flat surface, after exhaling, at a level parallel to the floor (waist circumference, hip circumference, height), using a stretch-resistant measuring tape to the nearest 1 mm. All anthropometric measures were measured to one decimal. Waist circumference was measured at the approximate midpoint between the lower margin of the last palpable rib and the top of the iliac crest. Hip circumference was measured around the widest portion of the buttocks. Body weight was measured using electronic calibrated scales to the nearest 100 grams, while subjects wore minimal clothing. Each measurement was repeated twice.

### 2.4. Statistical Analysis

Data analysis was performed using SAS software (version 8.02, Cary, NC, USA). Data in tables were reported with mean value and a standard deviation. Normality of distribution was tested using the Shapiro-Wilks test. Group comparisons were done using Mann-Whitney test and Student's *t*-test (CI of 95%). Correlation was used to explore the nature of relationships among variables. Curve estimation models were used to assess the relationships among anthropometric variables, EAT and SAT thickness. ROC analysis was used to determine predictive capabilities of anthropometric variables EAT and SAT thickness for CAD. Accepted statistical significance was for *p* < 0.05.

## 3. Results

Subjects were assigned to CAD group (*n* = 53) if they had ≥50% narrowing of one or more coronary arteries or to control, non-CAD group (*n* = 42), in which different valvular abnormalities were established. Subjects in the groups were of similar age and BMI. Larger values of waist circumference (WC), hip circumference (HC), waist-to-hip ratio (WHR), waist-to-height ratio (WHtR), EAT thickness, and SAT thickness were observed in CAD patients (Table 1).

Epicardial adipose tissue thickness showed weak to moderate correlation with WC and HC and weak correlation with BMI, WHtR, and SAT, while SAT showed stronger correlation with BMI, WC, HC, and WHtR. Neither EAT nor SAT correlated with age and WHR.

### 3.1. Nonlinear Relationships between the Variables

**Figure 1:**
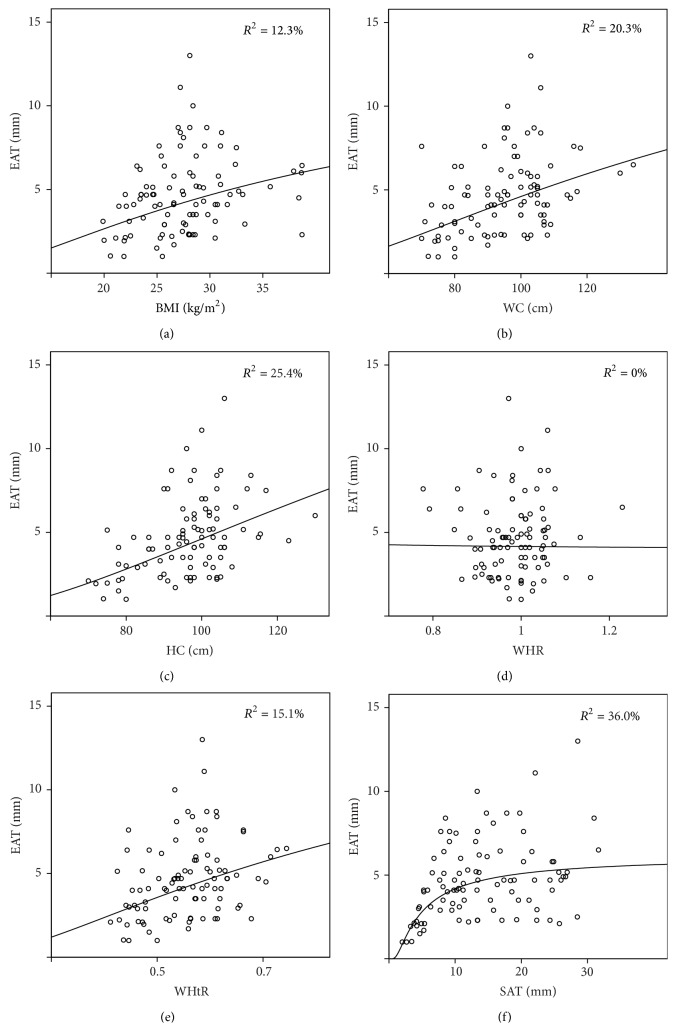
Nonlinear* S curve* relationship of EAT thickness with anthropometric measures and SAT thickness.

**Figure 2:**
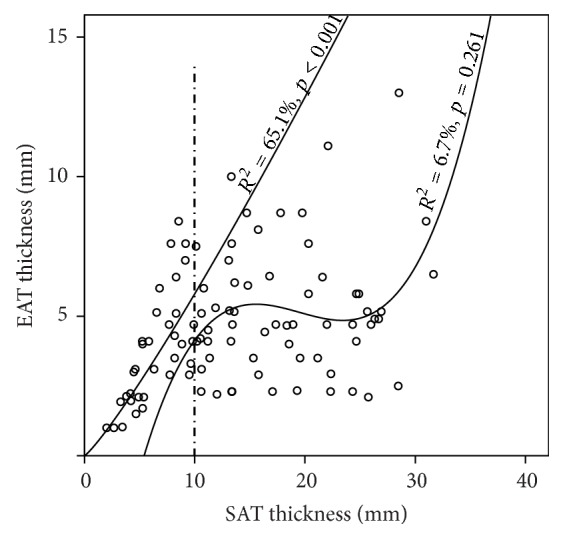
Nonlinear* power curve* relationship of EAT thickness and SAT thinner than 10 mm.

Relationship between anthropometric variables (except WHR) and EAT thickness was described the best as a nonlinear* S curve* relationship (Figures 1(a)–1(e)). An* S curve* model was also the best fit for EAT and SAT thicknesses' relationship (Figure 1(f)). Visual inspection of scatter plots revealed that SAT thinner than 10 mm had a different relationship with EAT than SAT thicker than 10 mm. Relationship between SAT thinner than 10 mm and EAT thickness was described the best as* power curve* relationship, since the* power curve* model explained 65.1% of variance (*p* < 0.001) (Figure 2). When SAT thickness was above 10 mm, no relationship with EAT thickness was established, as all regression models were nonsignificant (Figure 2).

### 3.2. Receiver Operating Characteristic Analysis

Receiver operating characteristic (ROC) curves for CAD classification with anthropometric variables and EAT, SAT thickness showed that all predictor variables, except BMI (*p* = 0.082), can detect CAD (*p* < 0.05), but with poor accuracy and similar sensitivity and specificity. Anthropometric variables and EAT and SAT had similar areas under curve. Using logistic regression for CAD prediction, overall model with anthropometric measures, SAT and EAT thickness as predictor variables were created and tested using ROC analysis. The overall model had moderate accuracy and the largest area under curve (AUC 0.751), while areas under curve for assessed measures ranged from 0.605 to 0.688 (Table 2).

## 4. Discussion

In our study cohort, all anthropometric variables, except WHR, showed moderate positive correlations with ultrasound measures of EAT and SAT. Such finding is obvious, since energy intake and expenditure disproportion lead to accumulation of body fat at all locations but also means that used anthropometric variables cannot discriminate between EAT and SAT. Lack of correlation and relationship of EAT and SAT with WHR might indicate that WHR is not valuable anthropometric index for prediction of EAT and SAT. It was also reported previously that several other anthropometric measures are superior for assessment of body fat distribution than WHR [13–15].

All anthropometric variables (except WHR) and SAT thickness, independently of each other, showed a nonlinear* S curve* relationship with EAT thickness in all analyzed subjects (Figure 1). As classic anthropometric measures measure both VAT and SAT, observed nonlinear relationship of EAT and classic anthropometric measures might be a “blurred picture” of an* S curve* clearly evident in relationship between EAT and SAT. Interestingly, in patients with SAT thinner than 10 mm, we observed strong nonlinear* power curve* relationship with EAT (explaining 65.1% of the variance), while SAT thicker than 10 mm is independent of EAT thickness, since we could not establish any linear or nonlinear relationship (Figure 2). This unexpected findings, along with the average maximum thickness of EAT (5.25 ± 2.50 mm), indicate that EAT does not accumulate above certain thickness regardless of increase in total adipose tissue volume.

Adipocytes in different compartments of adipose tissue enlarge by storing excess lipids, but when certain threshold overall volume of adipose tissue is reached, they cannot accumulate lipids anymore. It was shown that every person enters early adulthood with specific number of adipocytes, which can only be hypertrophy when accumulation of lipids occurs, while hyperplasia of precursors is strictly regulated [16]. Therefore, it is reasonable to predict that certain volume of adipose tissue has corresponding threshold values of different anthropometric measures.

Adipose tissue expands and undergoes extensive remodeling during positive caloric balance [16]. Adipose tissue remodeling occurs under inflammation and is characterized with relatively inadequate angiogenic remodeling and accumulation of extracellular matrix and immune cells [16]. It was reported that hypertrophy of the adipose tissue is associated with inability of recruitment and/or differentiation of existing preadipocytes into mature adipocytes, leading to overfill of adipocytes with lipids (lipid spillover) [17, 18]. Lipid spillover, through release of free fatty acids, acts as an inductor of endoplasmic stress and toll-like receptor type 4 pathways, responsible for the inflammation of the adipose tissue [19]. The enlargement of adipocytes leads to macrophage infiltration [17, 18, 20]. Curat et al. [21] showed,* in vitro*, that leptin (mostly produced by SAT) was responsible for macrophage extravasation, important step in initiation of adipose tissue inflammation [22]. Along with lipid spillover and leptin secretion, adipocyte hypertrophy is associated with hypoxia, a known inductor of inflammation [23]. Hypoxia acts through hypoxia induced factor 1, transcription factor that leads to extensive extracellular remodeling of adipose tissue, thus hampering angiogenesis, which is a rate limiting step in adipose tissue expansion [24]. Hypoxia occurs as adipocyte grows above 150–200 *μ*m in diameter, thus exciding oxygen diffusion distance [25]. These pathophysiological processes could explain observed nonlinear relationship of EAT and other measures, indicating that EAT undergoes remodeling and inflammation, thus hindering its ability to expand.

Moreover, accumulation of total adipose tissue leads to general low-grade inflammation, resulting in development of atherosclerosis [26]. Inflammation and atherosclerosis are the result of interplay of different cytokines, with important role of adipocytokines secreted by adipose tissue [26]. Coronarography is considered as standard for diagnosing CAD. Since it has several disadvantages and complications, complementary methods for assessing status of the coronary arteries would be useful. Anthropometric measures and SAT and EAT had poor accuracy at discriminating between CAD and controls in our study sample. The combined, overall model of anthropometric measures and SAT and EAT thickness had moderate accuracy at discriminating CAD and non-CAD patients. Since increased EAT thickness has been linked to CAD [3, 4, 11, 12], EAT thickness was expected to classify CAD patients better. Poor predictive accuracy of EAT thickness for CAD might be explained with current study setting, where control group consisted of patients with valvular diseases as controls. Recently, atherosclerotic pathophysiological process that results in CAD was associated with the aortic stenosis, the most common valvular disease [27]. Visceral obesity and its proinflammatory adipocytokines and subsequent inflammation have been linked with aortic stenosis, so called valvulometabolic risk [28]. Decreased levels of anti-inflammatory adiponectin have been linked with greater valvular inflammatory activity [29], while increased levels of leptin have been found in blood of the valvular patients [30]. Systemic effects of different compartments of adipose tissue could contribute to development of aortic stenosis and other valvular disease, as well as potential paracrine effects of the EAT and periaortic adipose tissue. Therefore, similar pathophysiological background of CAD and valvular disease might explain poor predictive accuracy of EAT thickness in our study sample, where the proportion of our control patients had aortic stenosis. Nevertheless, further research on role of EAT is warranted, since, in healthy individuals, it protects the myocardium while its expansion might lead to lipotoxicity and generation of proinflammatory cytokines [31]. Imaging MRI and echocardiographic studies showed that myocardial fat and cardiac dysfunction (i.e., left ventricle LV overload, systolic dysfunction, and hypertrophy) are associated with accumulation of EAT [32, 33]. Our study is limited to lack of expression profile of inflammation markers in adipose tissue and blood, as well as a relatively small sample size.

In conclusion, in cardiac patients, the EAT thickness follows a nonlinear relationship with anthropometric measures and SAT thickness. Ultrasound measures of EAT thickness have similar predictive accuracy for CAD to anthropometric measures of visceral obesity.

## Figures and Tables

**Table 1:** Anthropometric measures and EAT and SAT thickness in CAD and non-CAD subjects.

	CAD	Non-CAD
Number	53	42
Age (years)	62 ± 10	62 ± 13
BMI (kg/m^2^)	27.90 ± 3.08	27.03 ± 5.30
Waist circumference (cm)	98.84 ± 12.19	89.95 ± 12.82^∗^
Hip circumference (cm)	98.96 ± 9.39	93.32 ± 12.59^∗^
Waist-to-hip ratio	0.99 ± 0.08	0.96 ± 0.05^∗^
Waist-to-height ratio	0.57 ± 0.06	0.53 ± 0.08^∗^
EAT thickness (mm)	5.25 ± 2.50	3.89 ± 1.76^∗^
SAT thickness (mm)	15.38 ± 6.67	12.32 ± 8.33^∗^

CAD: coronary artery disease; BMI: body mass index; EAT: epicardial adipose tissue; SAT: subcutaneous adipose tissue. ^∗^ *p* < 0.05: difference between the groups.

**Table 2:** Receiver operating characteristic (ROC) analysis for prediction of coronary artery disease (CAD) with anthropometric measures and ultrasonographically obtained epicaradial and subcutaneous adipose tissue thickness.

Tested variable	AUC	*p*	95% CI
BMI	0.605	0.082	0.481–0.729
Waist circumference	0.688	0.002	0.579–0.797
Hip circumference	0.643	0.018	0.528–0.758
Waist-to-hip ratio	0.657	0.009	0.545–0.770
Waist-to-height ratio	0.673	0.004	0.559–0.786
EAT thickness	0.658	0.009	0.548–0.768
SAT thickness	0.634	0.027	0.511–0.756

Overall model	0.751	<0.001	0.651–0.834

Overall logistic regression model for CAD classification with anthropometric measures and EAT and SAT as predictor variables was tested with ROC analysis. AUC: area under curve; CI: confidence interval; BMI: body mass index; EAT: epicardial adipose tissue; SAT: subcutaneous adipose tissue. Coronary artery disease (CAD) was defined as ≥50% narrowing of one or more arteries.
